# Supplementary material for: Ep400 deficiency in Schwann cells causes persistent expression of early developmental regulators and peripheral neuropathy
Source: Nat Commun. 2019 May 29;10:2361. doi: 10.1038/s41467-019-10287-w (PMC6541636; doi:10.1038/s41467-019-10287-w)
Supplement: Supplementary file 1 — Supplementary Information [file 41467_2019_10287_MOESM1_ESM.pdf]

Supplementary Information

for

Ep400 deficiency in Schwann cells causes persistent expression of early developmental regulators and peripheral neuropathy

by

Franziska Fröb et al.

SUPPLEMENTARY FIGURES

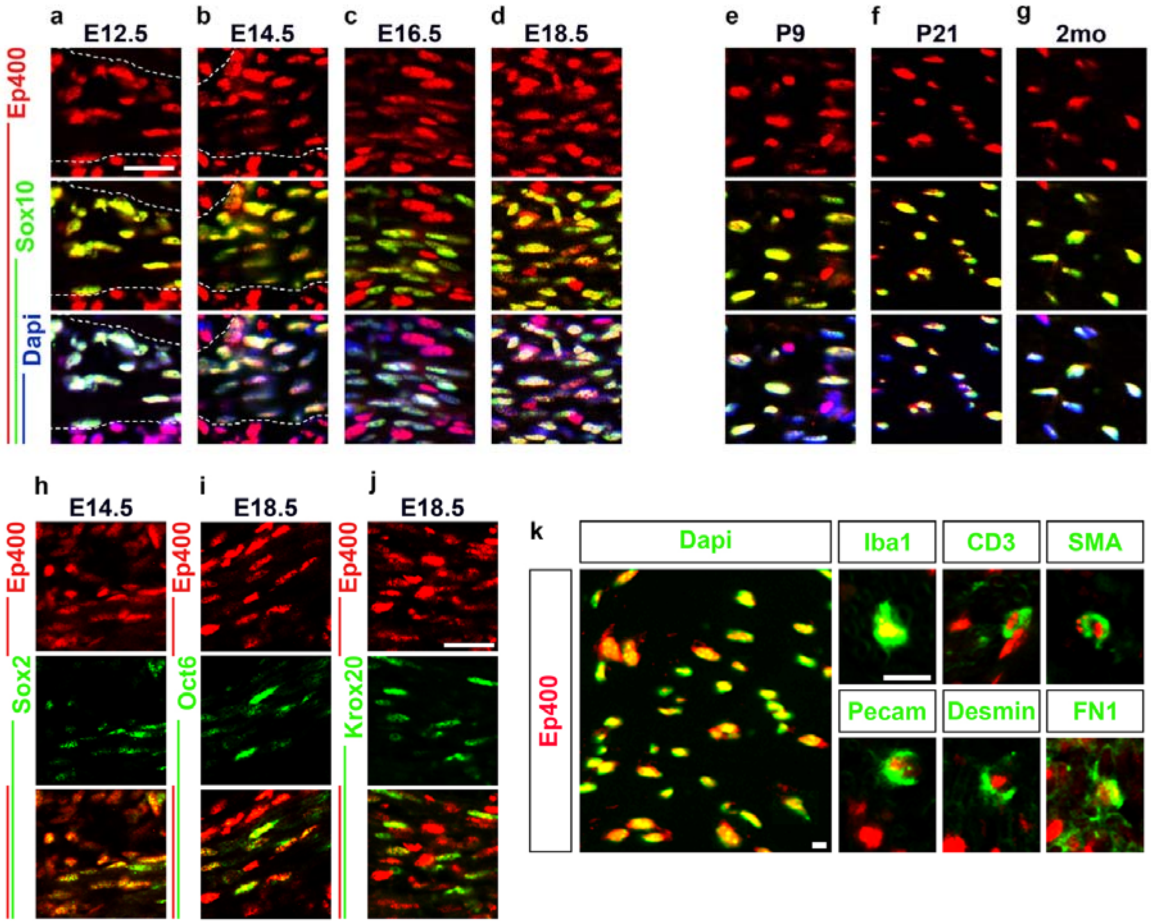

Supplementary Figure 1: Ep400 expression in Schwann cells. (**a-g**) Localization of Ep400 (red) in Sox10-labeled (green) Schwann cells (SCs) by immunofluorescence studies on spinal nerve (a-d) and sciatic nerve (e-g) sections at E12.5 (a), E14.5 (b), E16.5 (c), E18.5 (d), P9 (e), P21 (f) and 2 months (2mo, g) of age. Nuclei were counterstained with DAPI (blue). (**h-j**) Co-localization of Ep400 (red) with the stage-specific SC markers Sox2 (h), Oct6 (i) and Krox20 (j) (all in green) by immunohistochemistry on spinal nerve sections at E14.5 (h), and E18.5 (i,j). (**k**) Localization of Ep400 (red) in other cell types of the sciatic nerve at P9 such as Iba1-positive macrophages, CD3-positive T lymphocytes, α-Smooth muscle actin (Sma) positive perivascular smooth muscle cells, Pecam-positive endothelial cells, Desmin-positive pericytes and fibronectin (FN1) expressing fibroblasts. Cell-type specific markers and nuclear DAPI stain are in green. Scale bars: 25µm (a,j), 10µm (k).

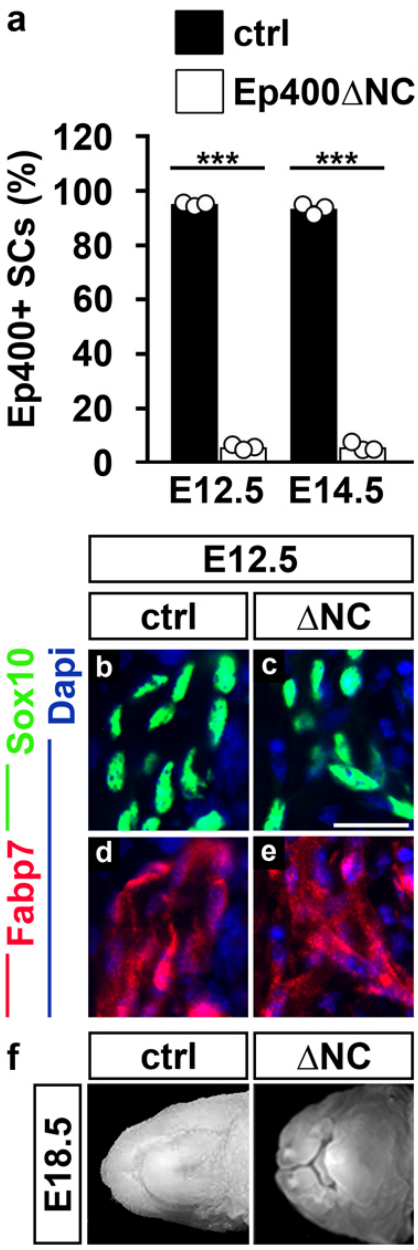

Supplementary Figure 2: Early neural-crest specific deletion of Ep400. (**a**) Ep400 deletion in SC precursors along spinal nerves of *Ep400ΔNC* mice at E12.5 and E14.5 as determined by Ep400 immunohistochemistry relative to age-matched controls. SCs (SC) were identified by Sox10 staining (n = 3; mean values ± SEM). Statistical significance was determined by unpaired two-tailed Student's t-test (***, P ≤0.001). (**b-e**) Presence of SC precursors along spinal nerves of control and *Ep400ΔNC* mice at E12.5 as determined by immunohistochemical detection of Sox10 (green) (b,c) and Fabp7 (red) (d,e). Nuclei were counterstained with DAPI (blue). Scale bars: 25µm (c). (**f**) Ventral view of head from control and *Ep400ΔNC* embryos at E18.5 showing orofacial clefting in the mutant. Exact values are listed in Suppl. Table 10 and source data are provided as a Source Data file.

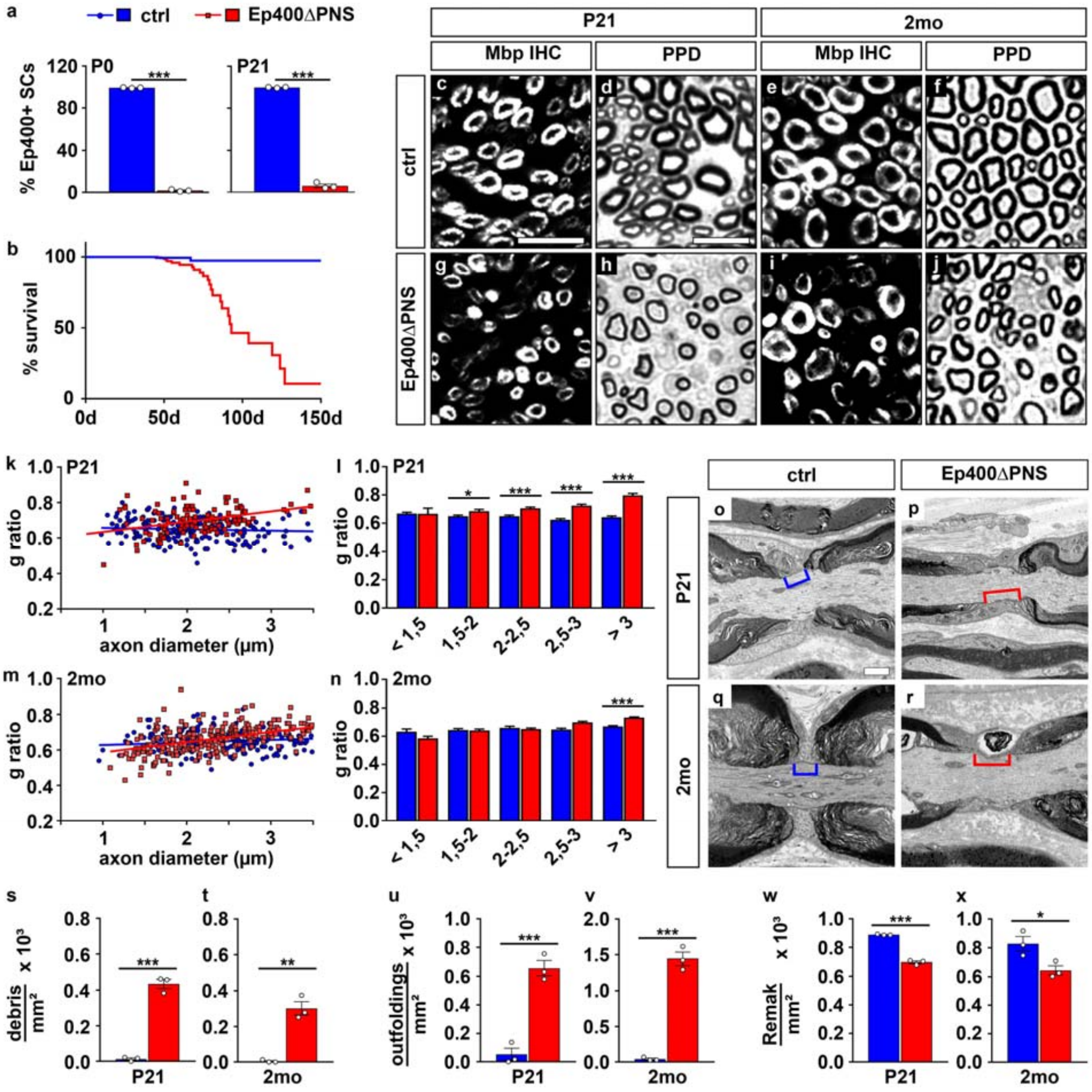

Supplementary Figure 3: Myelination defects in *Ep400ΔPNS* mice. (**a**) Ep400 deletion in sciatic nerve SCs of *Ep400ΔPNS* mice (red bars) at P0 and P21 as determined by Ep400 immunohistochemistry relative to age-matched controls (blue bars). SCs were identified by Sox10 staining (n = 3; mean values ± SEM). (**b**) Survival curve of control (blue line) and *Ep400ΔPNS* (red line) mice during the first 5 months of life. (**c-j**) Myelin structures visualized by Mbp immunohistochemistry (c,e,g,i) and PPD staining (d,f,h,j) on transverse sciatic nerve sections of control (c-f) and *Ep400ΔPNS* (g-j) mice at P21 (c,d,g,h) and 2mo (e,f,i,j). (**k-n**) Determination of single g ratios (scatter plots in k,m) and g ratio in relation to axon size after binning of axons according to their diameter (l,n) from ultrathin sciatic nerve sections of control (blue) and *Ep400ΔPNS* (red) mice at P21 (k,l) and 2 months (2mo) of age (m,n) using n = 100-300 axons for each age (mean g ratios ± SEM). (**o-r**) Appearance of node and paranode on ultrathin longitudinal sections of sciatic nerves from control (o,q) and *Ep400ΔPNS* (p,r) mice at P21 (o,p) and 2 months (q,r) of age. (**s-x**) Quantification of myelin

debris (s,t), myelin outfoldings (u,v) and Remak bundles (w,x) on ultrathin transverse sections of sciatic nerves from control (blue) and *Ep400ΔPNS* (red) mice at P21 (s,u,w) and 2 months (t,v,x). Statistical significance was determined by unpaired, two-tailed Student's t-test (*, $P \leq 0.05$; **, $P \leq 0.01$; ***, $P \leq 0.001$). Scale bars: 25µm (c), 1µm (o). Exact values are listed in Suppl. Table 1,9,10 and source data are provided as a Source Data file.

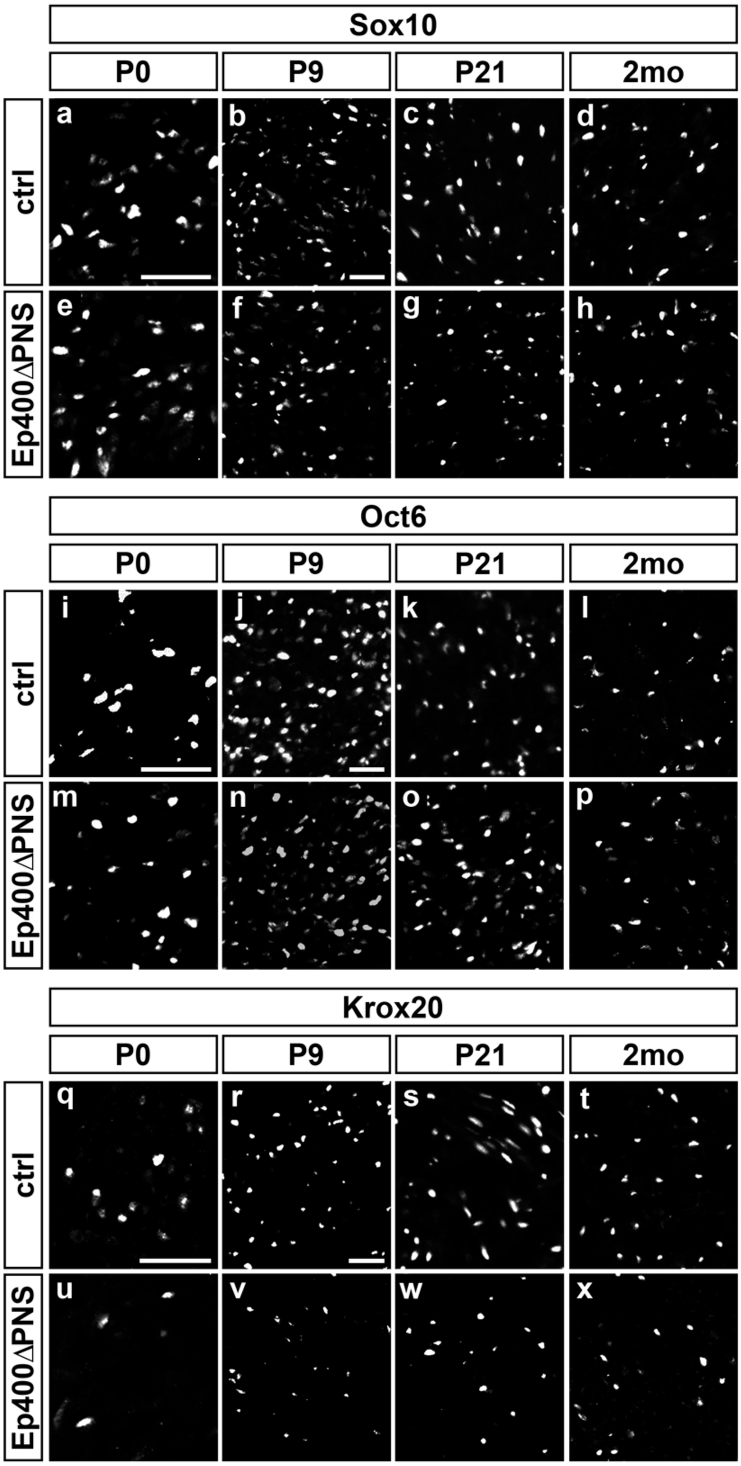

Supplementary Figure 4: Expression of Schwann cell regulatory factors in *Ep400ΔPNS* mice. (**a-x**) Immunohistochemical stainings of sciatic nerve sections of control (a-d,i-l,q-t) and *Ep400ΔPNS* (e-h,m-p,u-x) mice at P0 (a,e,i,m,q,u), P9 (b,f,j,n,r,v), P21 (c,g,k,o,s,w) and 2months (2mo) (d,h,l,p,t,x) with antibodies directed against Sox10 (a-h), Oct6 (i-p) and Krox20 (q-x). Scale bars: 25µm.

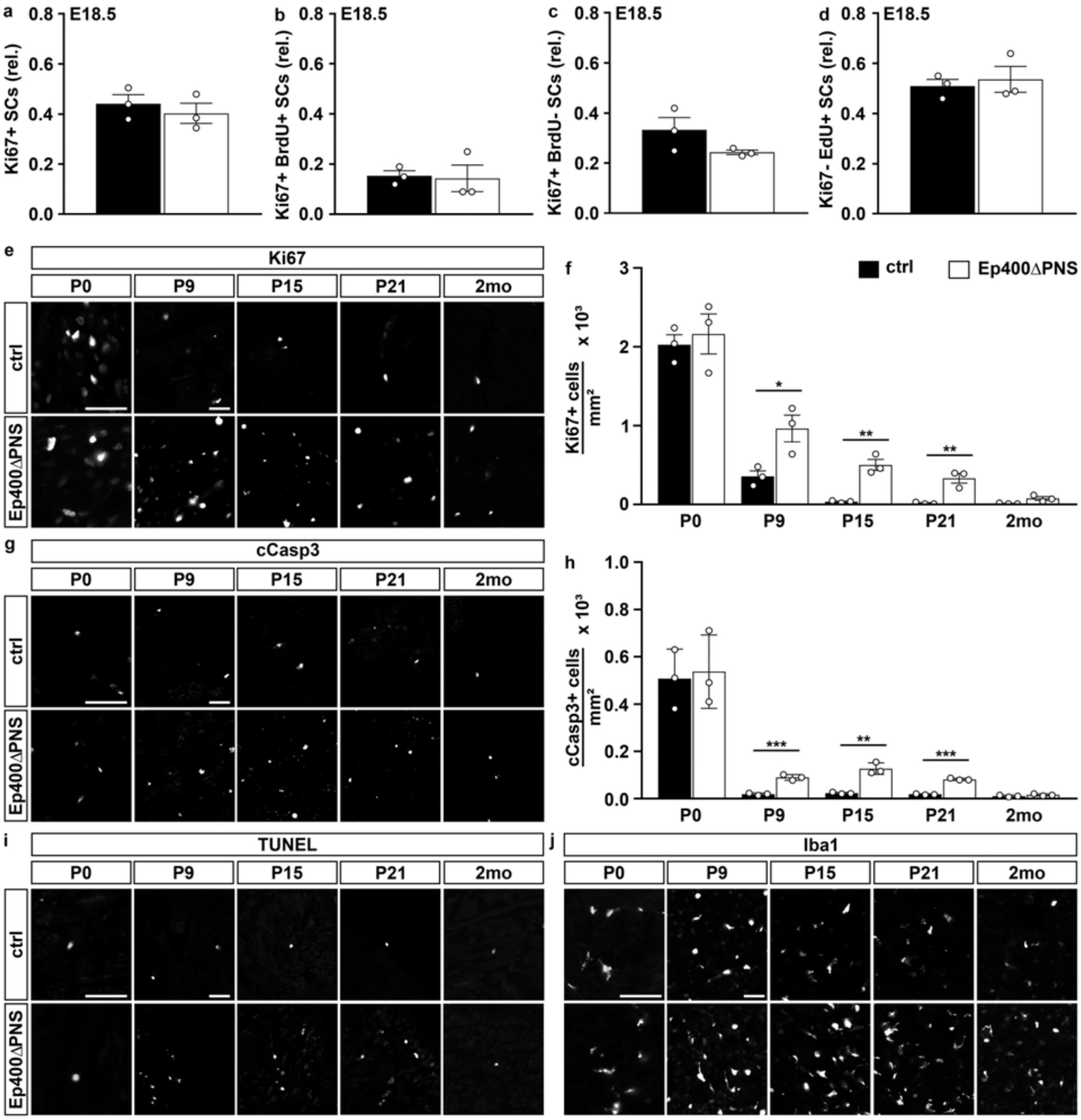

Supplementary Figure 5: General and cell type-specific changes of proliferation, apoptosis and inflammation in sciatic nerves of in *Ep400ΔPNS* mice. (**a-d**) Quantification of SC proliferation at E18.5 after treating pregnant mice at E17.5 with EdU and 1 h before harvesting the embryos by caesarean section with BrdU. The fraction of SCs in cell cycle was determined as the fraction of Ki67-positive SCs (a), the actively dividing fraction as the fraction of Ki67- and BrdU-positive SCs (b), the non-dividing fraction as the percentage of Ki67-positive, but BrdU-negative SCs (c), and the fraction that had exited the cell cycle during the last 24h as the percentage of Ki67-negative SCs among the ones labelled with EdU one day prior to analysis (d) in spinal nerves of control (black bars) and *Ep400ΔPNS* (white bars) embryos (n = 3 each, mean values ± SEM). (**e-j**) Immunohistochemical stainings with antibodies directed against Ki67 (e), cleaved Caspase 3 (g) and Iba1 (j) or TUNEL (i) of sciatic nerve sections of control and *Ep400ΔPNS* mice at P0, P9, P15, P21 and 2 months

(2mo) and corresponding quantifications of Ki67-positive (f) and cleaved Caspase 3-positive (h) cells (n = 3; mean values ± SEM). Scale bars: 25µm. Statistical significance was determined by two-tailed Student's t-test (*, P ≤0.05; **, P ≤0.01; ***, P ≤0.001). Exact values are listed in Suppl. Table 5,11 and source data are provided as a Source Data file.

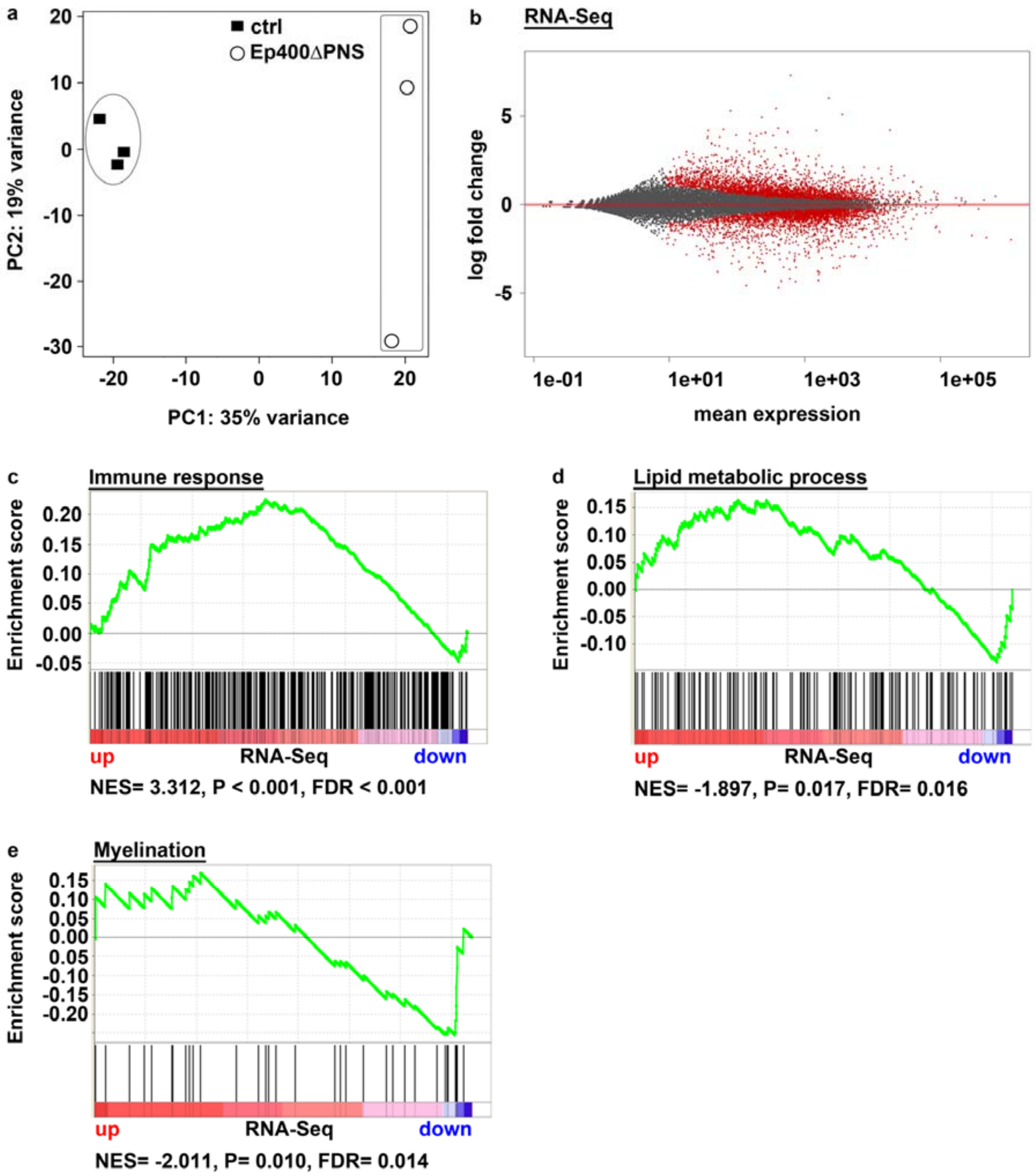

Supplementary Figure 6: RNA-Seq data from sciatic nerves of control and *Ep400ΔPNS* mice. (**a**) Principal component analysis blot of RNA-Seq samples from sciatic nerves of control (black squares) and *Ep400ΔPNS* (white circles) mice at P9. (**b**) MA plot depicting the overall gene expression changes (red dots) in sciatic nerves of *Ep400ΔPNS* mice relative to controls. (**c-e**) Gene set enrichment blots from GSEA analysis of RNA-Seq data with the following gene sets: immune response (c), lipid metabolic process (d) and myelination (e). Normalized enrichment score (NES), p value and false discovery rate (FDR) are listed at the bottom of each blot.

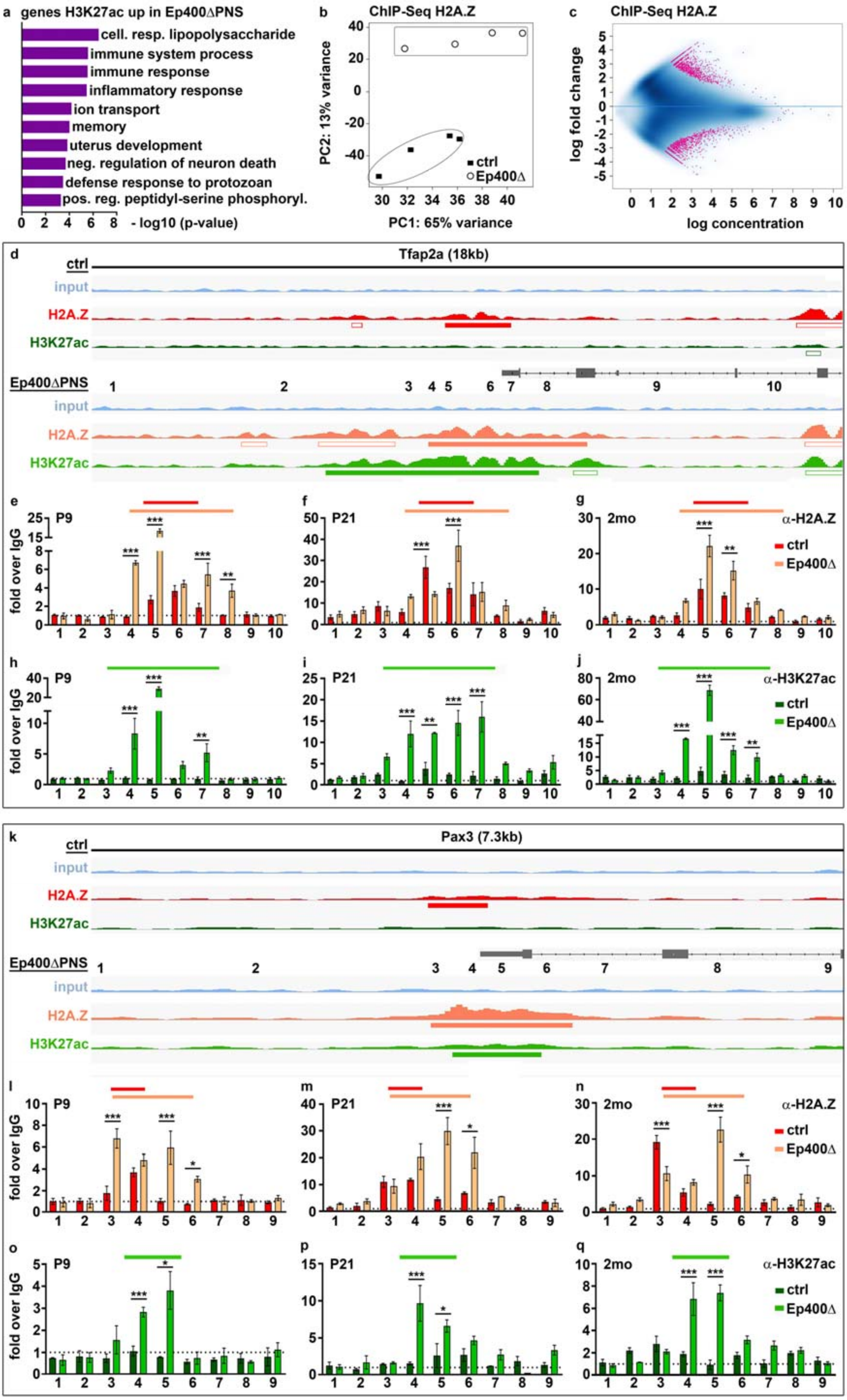
a genes H3K27ac up in Ep400ΔPNS
cell. resp. lipopolysaccharide
immune system process
immune response
inflammatory response
ion transport
memory
uterus development
neg. regulation of neuron death
defense response to protozoan
pos. reg. peptidyl-serine phosphoryl.
- log10 (p-value)
b ChIP-Seq H2A.Z
PC2: 13% variance
PC1: 65% variance
ctrl
Ep400Δ
c ChIP-Seq H2A.Z
log fold change
log concentration
d Tfap2a (18kb)
ctrl
input
H2A.Z
H3K27ac
Ep400ΔPNS
e P9
f P21
g 2mo
α-H2A.Z
ctrl
Ep400Δ
fold over IgG
h P9
i P21
j 2mo
α-H3K27ac
k Pax3 (7.3kb)
l P9
m P21
n 2mo
o P9
p P21
q 2mo

Supplementary Figure 7: ChIP data from sciatic nerves of control and *Ep400ΔPNS* mice. (**a**) GO analysis (DAVID) for biological processes enriched among genes that gained promoter-associated H3K27ac in *Ep400ΔPNS* nerves. Processes are sorted by statistical significance. (**b**) Principal component analysis blot of ChIP-Seq from H2A.Z samples from sciatic nerves of control (black squares) and *Ep400ΔPNS* (white circles) mice at P9. (**c**) MA plot depicting the overall differences (red dots) in affinity to binding sites in sciatic nerves of *Ep400ΔPNS* mice relative to controls. (**d-q**) Genomic distribution of H2A.Z (d-g,k-n) and H3K27ac (d,h-j,k,o-q) in chromatin from sciatic nerves of control (red, dark green) and *Ep400ΔPNS* (orange, light green) mice in the *Tfap2a* (d-j) and *Pax3* (k-q) genomic loci at P9 (d,e,h,k,l,o), P21 (f,i,m,p) and 2 months (g,j,n,q). Data are from ChIP-Seq (d,k) and ChIP-qPCR (e-j,l-q) experiments. ChIP-Seq data are presented as tracks and are expanded relative to those shown in Fig. 4f,g. TSS-associated peaks for H2A.Z are marked by red (control) or orange (*Ep400ΔPNS*) lines below the tracks, those for H3K27ac by dark (control) or light (*Ep400ΔPNS*) green lines. Other peaks are marked by lines with coloured rim. For ChIP-qPCR experiments, several regions distributed throughout the *Tfap2a* and *Pax3* loci (for position, see numbers in d,k) were analyzed by quantitative PCR for enrichment in chromatin from sciatic nerves of control (red and dark green bars) and *Ep400ΔPNS* (orange and light green bars) mice after precipitation with antibodies directed against H2A.Z and H3K27ac compared to IgG control (arbitrarily set to 1, dotted line). ChIP-qPCR data are presented as mean values ± SEM (n = 3). The relative position of TSS-associated peaks from ChIP-Seq studies to the regions analyzed in ChIP-qPCR is indicated by coloured horizontal lines at the top of each ChIP-qPCR panel. Statistical significance was determined by two-tailed Student's t-test (*, $P \leq 0.05$; **, $P \leq 0.01$; ***, $P \leq 0.001$). Exact values and primer sequences for ChIP-qPCR experiments are listed in Suppl. Tables 12,13 and source data are provided as a Source Data file.

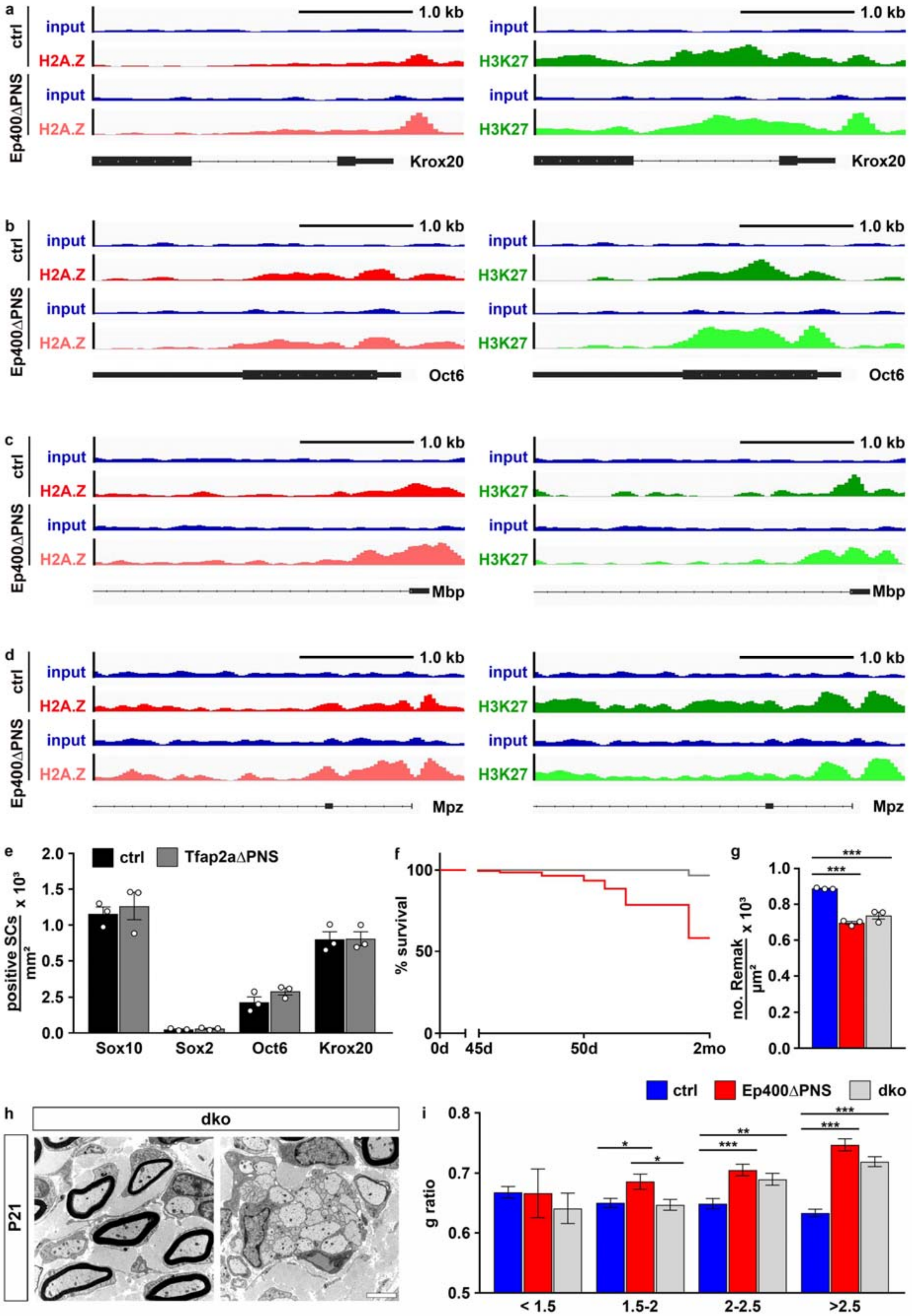

a
b
c
d
ctrl
Ep400ΔPNS
input
H2A.Z
H3K27
1.0 kb
Krox20
Oct6
Mbp
Mpz
e
ctrl
Tfap2aΔPNS
positive SCs / mm² x 10³
Sox10
Sox2
Oct6
Krox20
f
% survival
0d
45d
50d
2mo
g
no. Remak / µm² x 10³
h
dko
P21
i
ctrl
Ep400ΔPNS
dko
g ratio
< 1.5
1.5-2
2-2.5
>2.5

Supplementary Figure 8: Characterization of chromatin at select promoters in *Ep400ΔPNS* mice and Schwann cell phenotype in dko mice. (**a-d**) Tracks from H2A.Z (left) and H3K27ac (right) ChIP-Seq experiments for *Krox20* (a), *Oct6 (b), Mbp* (c) and *Mpz* (d) performed on chromatin from sciatic nerves of control (red and dark green) and *Ep400ΔPNS* (orange and light green) mice at P9. Shown are H2A.Z precipitates, H3K27ac precipitates and input (blue) for both genotypes. Differential peaks were not detected and would be marked by colored lines below the corresponding tracks. (**e**) Comparison of the total SC number and the number of Sox2-, Oct6-, and Krox20- positive SCs in sciatic nerve sections of control (black bars) and *Tfap2aΔPNS* (dark grey bars) mice at P21 (n = 3; mean values ± SEM). (**f**) Survival curve of *Ep400ΔPNS* (red line) and dko (light grey line) mice during the first 2 months of life. (**g**) Quantification of Remak bundles on ultrathin transverse sections of sciatic nerves from control (blue bars), *Ep400ΔPNS* (red bars) and dko (light grey bars) mice at P21 (n = 3; mean values ± SEM). Values for control and *Ep400ΔPNS* mice are those shown in Suppl. Fig. 3w. (**h**) Representative electron microscopic pictures of sciatic nerve sections from dko mice at P21. For comparison with control and *Ep400ΔPNS* mice, see Fig. 1i-l. Scale bar: 2.5µm. (**i**) Determination of g ratio in relation to axon size after binning of axons according to their diameter from ultrathin sciatic nerve sections of control (blue bars), *Ep400ΔPNS* (red bars) and dko (light grey bars) mice at P21 (n = 100-200 axons). Values for control and *Ep400ΔPNS* mice are those shown in Suppl. Fig. 3l. Statistical significance was determined by two-tailed Student’s t-test (e) or ANOVA (g,i) (*, P ≤0.05; **, P ≤0.01; ***, P ≤0.001). Exact values are listed in Suppl. Tables 1,5,9 and source data are provided as a Source Data file.

SUPPLEMENTARY TABLES

Supplementary Table 1: G ratio of myelin sheaths in sciatic nerves of control and mutant mice

Age	Genotype	Mean value ± SEM	Figure
P21	control	0.65 ± 0.01	Figs. 1q & 5n
	<1.5µm Ø axons	0.67 ± 0.01	Suppl. Figs. 3l & 8i
	1.5-2µm Ø axons	0.65 ± 0.01	Suppl. Figs. 3l & 8i
	2-2.5µm Ø axons	0.65 ± 0.01	Suppl. Figs. 3l & 8i
	2.5-3µm Ø axons	0.62 ± 0.01	Suppl. Figs. 3l & 8i
	>3µm Ø axons	0.64 ± 0.01	Suppl. Figs. 3l
	>2.5µm Ø axons	0.63 ± 0.01	Suppl. Figs. 8i
	Ep400ΔPNS	0.70 ± 0.01	Figs. 1q & 5n
	<1.5µm Ø axons	0.67 ± 0.04	Suppl. Figs. 3l & 8i
	1.5-2µm Ø axons	0.69 ± 0.01	Suppl. Figs. 3l & 8i
	2-2.5µm Ø axons	0.71 ± 0.01	Suppl. Figs. 3l & 8i
	2.5-3µm Ø axons	0.72 ± 0.01	Suppl. Figs. 3l & 8i
	>3µm Ø axons	0.80 ± 0.01	Suppl. Figs. 3l
	>2.5µm Ø axons	0.75 ± 0.01	Suppl. Figs. 8i
	dko	0.67 ± 0.01	Fig. 5n
	<1.5µm Ø axons	0.64 ± 0.03	Suppl. Fig. 8i
	1.5-2µm Ø axons	0.65 ± 0.01	Suppl. Fig. 8i
	2-2.5µm Ø axons	0.69 ± 0.01	Suppl. Fig. 8i
	>2.5µm Ø axons	0.72 ± 0.01	Suppl. Fig. 8i
2 months	control	0.66 ± 0.01	Fig. 1u
	<1.5µm Ø axons	0.63 ± 0.02	Suppl. Fig. 3n
	1.5-2µm Ø axons	0.64 ± 0.01	Suppl. Fig. 3n
	2-2.5µm Ø axons	0.66 ± 0.01	Suppl. Fig. 3n
	2.5-3µm Ø axons	0.65 ± 0.01	Suppl. Fig. 3n
	>3µm Ø axons	0.67 ± 0.01	Suppl. Fig. 3n
	Ep400ΔPNS	0.67 ± 0.01	Fig. 1u
	<1.5µm Ø axons	0.58 ± 0.02	Suppl. Fig. 3n
	1.5-2µm Ø axons	0.64 ± 0.01	Suppl. Fig. 3n
	2-2.5µm Ø axons	0.65 ± 0.01	Suppl. Fig. 3n
	2.5-3µm Ø axons	0.70 ± 0.01	Suppl. Fig. 3n
	>3µm Ø axons	0.73 ± 0.01	Suppl. Fig. 3n

Supplementary Table 2: Percentage of myelinated large caliber axons in sciatic nerves of control and mutant mice

Age	Genotype	Mean value ± SEM	Figure
P21	control	100 ± 0.00 %	Fig. 1r
	Ep400ΔPNS	74.67 ± 2.33%	Fig. 1r
2 months	control	100 ± 0.00 %	Fig. 1v
	Ep400ΔPNS	97.00 ± 1.00%	Fig. 1v

Supplementary Table 3: Relative length distribution of internodes in sciatic nerves of control and mutant mice at P21

Genotype	Internodal length	Mean value ± SEM	Figure
control	0-50 µm	9.70 ± 4.85	Fig. 2a
	50-150 µm	58.78 ± 11.30	Fig. 2a
	150-200µm	27.82 ± 4.72	Fig. 2a
	200-250µm	3.70 ± 3.70	Fig. 2a
Ep400ΔPNS	0-50 µm	54.39 ± 8.15	Fig. 2a
	50-150 µm	48.82 ± 9.20	Fig. 2a
	150-200µm	0.00 ± 0.00	Fig. 2a
	200-250µm	0.00 ± 0.00	Fig. 2a

Supplementary Table 4: Length of nodes and paranodes in sciatic nerves of control and mutant mice at P21

Structure	Genotype	Mean value ± SEM	Figure
node	control	1.00 ± 0.02µm	Figs. 2c & 5q
	Ep400ΔPNS	1.34 ± 0.03µm	Figs. 2c & 5q
	dko	1.32 ± 0.08µm	Fig. 5q
paranode	control	4.28 ± 0.16µm	Figs. 2d & 5r
	Ep400ΔPNS	3.46 ± 0.13µm	Figs. 2d & 5r
	dko	3.66 ± 0.10µm	Fig. 5r

Supplementary Table 5: Number of marker-positive cells per defined area in sciatic nerves of control and mutant mice

Marker	Age	Genotype	Mean value ± SEM	Figure
Sox10	P0	control	8237 ± 440	Fig. 2e
		control	7800 ± 64	Fig. 5a
		Ep400ΔPNS	7630 ± 658	Fig. 2e
		Ep400ΔPNS	7950 ± 330	Fig. 5a
		dko	7943 ± 170	Fig. 5a
	P9	control	4340 ± 456	Fig. 2e
		control	3903 ± 47	Fig. 5a
		Ep400ΔPNS	4497 ± 739	Fig. 2e
		Ep400ΔPNS	4027 ± 15	Fig. 5a
		dko	4043 ± 232	Fig. 5a
	P21	control	1777 ± 100	Fig. 2e
		control	1656 ± 96	Fig. 5a, Suppl. Fig. 8e
		Ep400ΔPNS	1863 ± 103	Fig. 2e
		Ep400ΔPNS	1768 ± 294	Fig. 5a
		Tfap2aΔPNS	1766 ± 191	Suppl. Fig. 8e
		dko	1588 ± 120	Fig. 5a
	2 months	control	847 ± 98	Fig. 2e
		control	957 ± 19	Fig. 5a
		Ep400ΔPNS	850 ± 148	Fig. 2e
		Ep400ΔPNS	941 ± 16	Fig. 5a
		dko	944 ± 39	Fig. 5a
Oct6	P0	control	5677 ± 386	Fig. 2f
		control	5607 ± 124	Fig. 5c
		Ep400ΔPNS	5307 ± 94	Fig. 2f
		Ep400ΔPNS	5493 ± 50	Fig. 5c
		dko	5567 ± 284	Fig. 5c
	P9	control	3317 ± 147	Fig. 2f
		control	2891 ± 150	Fig. 5c
		Ep400ΔPNS	2857 ± 391	Fig. 2f
		Ep400ΔPNS	2747 ± 136	Fig. 5c
		dko	2780 ± 5	Fig. 5c
	P21	control	710 ± 150	Fig. 2f
		control	428 ± 76	Fig. 5c, Suppl. Fig.8e
		Ep400ΔPNS	1423 ± 163	Fig. 2f
		Ep400ΔPNS	1208 ± 143	Fig. 5c
		Tfap2aΔPNS	577 ± 45	Suppl. Fig.8e

		dko	879 ± 152	Fig. 5c
	2 months	control	310 ± 25	Fig. 2f
		control	194 ± 6	Fig. 5c
		Ep400ΔPNS	517 ± 99	Fig. 2f
		Ep400ΔPNS	339 ± 31	Fig. 5c
		dko	233 ± 8	Fig. 5c
Krox20	P0	control	2323 ± 277	Fig. 2g
		control	2343 ± 63	Fig. 5d
		Ep400ΔPNS	760 ± 284	Fig. 2g
		Ep400ΔPNS	910 ± 26	Fig. 5d
		dko	1577 ± 73	Fig. 5d
	P9	control	1630 ± 213	Fig. 2g
		control	1608 ± 34	Fig. 5d
		Ep400ΔPNS	480 ± 56	Fig. 2g
		Ep400ΔPNS	527 ± 25	Fig. 5d
		dko	1088 ± 64	Fig. 5d
	P21	control	1893 ± 42	Fig. 2g
		control	1301 ± 107	Fig. 5d, Suppl. Fig.8e
		Ep400ΔPNS	903 ± 153	Fig. 2g
		Ep400ΔPNS	791 ± 16	Fig. 5d
		Tfap2aΔPNS	1310 ± 97	Suppl. Fig.8e
		dko	1142 ± 13	Fig. 5d
	2 months	control	880 ± 114	Fig. 2g
		control	841 ± 39	Fig. 5d
		Ep400ΔPNS	750 ± 95	Fig. 2g
		Ep400ΔPNS	744 ± 36	Fig. 5d
		dko	826 ± 9	Fig. 5d
Sox2	P0	control	2203 ± 227	Fig. 5b
		Ep400ΔPNS	1577 ± 191	Fig. 5b
		dko	2080 ± 391	Fig. 5b
	P9	control	65 ± 4	Fig. 5b
		Ep400ΔPNS	349 ± 11	Fig. 5b
		dko	211 ± 23	Fig. 5b
	P21	control	51 ± 8	Fig. 5b, Suppl. Fig.8e
		Ep400ΔPNS	347 ± 71	Fig. 5b
		Tfap2aΔPNS	61 ± 9	Suppl. Fig. 8e
		dko	140 ± 21	Fig. 5b
	2 months	control	54 ± 3	Fig. 5b
		Ep400ΔPNS	98 ± 1	Fig. 5b
		dko	52 ± 3	Fig. 5b
Mbp	P21	control	57 ± 4	Fig. 5e
		Tfap2aΔPNS	53 ± 1	Fig. 5e

		Ep400ΔPNS	27 ± 1	Fig. 5e
		dko	43 ± 4	Fig. 5e
Ki67 + Sox10	P0	control	1286 ± 116	Fig. 2h
		control	1278 ± 90	Fig. 5s
		Ep400ΔPNS	1317 ± 122	Fig. 2h
		Ep400ΔPNS	1254 ± 88	Fig. 5s
		dko	1292 ± 25	Fig. 5s
	P9	control	72 ± 4	Fig. 2h
		control	62 ± 2	Fig. 5s
		Ep400ΔPNS	323 ± 69	Fig. 2h
		Ep400ΔPNS	335 ± 27	Fig. 5s
		dko	191 ± 19	Fig. 5s
	P15	control	13 ± 6	Fig. 2h
		Ep400ΔPNS	184 ± 5	Fig. 2h
	P21	control	0 ± 0	Fig. 2h
		control	18 ± 1	Fig. 5s
		Ep400ΔPNS	161 ± 8	Fig. 2h
		Ep400ΔPNS	341 ± 29	Fig. 5s
		dko	154 ± 22	Fig. 5s
	2 months	control	0 ± 0	Fig. 2h
		control	16 ± 5	Fig. 5s
		Ep400ΔPNS	56 ± 9	Fig. 2h
		Ep400ΔPNS	78 ± 1	Fig. 5s
		dko	25 ± 6	Fig. 5s
Cleaved caspase 3 + Sox10	P0	control	234 ± 21	Fig. 2i
		control	219 ± 29	Fig. 5t
		Ep400ΔPNS	233 ± 31	Fig. 2i
		Ep400ΔPNS	211 ± 9	Fig. 5t
		dko	219 ± 7	Fig. 5t
	P9	control	20 ± 1	Fig. 2i
		control	18 ± 2	Fig. 5t
		Ep400ΔPNS	40 ± 4	Fig. 2i
		Ep400ΔPNS	44 ± 2	Fig. 5t
		dko	25 ± 2	Fig. 5t
	P15	control	7 ± 1	Fig. 2i
		Ep400ΔPNS	16 ± 3	Fig. 2i
	P21	control	0 ± 0	Fig. 2i
		control	0 ± 0	Fig. 5t
		Ep400ΔPNS	23 ± 2	Fig. 2i
		Ep400ΔPNS	16 ± 3	Fig. 5t
		dko	2 ± 2	Fig. 5t
	2 months	control	0 ± 0	Fig. 2i

		control	9 ± 1	Fig. 5t
		Ep400ΔPNS	0 ± 0	Fig. 2i
		Ep400ΔPNS	14 ± 2	Fig. 5t
		dko	11 ± 3	Fig. 5t
TUNEL	P0	control	136 ± 8	Fig. 2j
		Ep400ΔPNS	122 ± 11	Fig. 2j
	P9	control	41 ± 1	Fig. 2j
		Ep400ΔPNS	71 ± 4	Fig. 2j
	P15	control	15 ± 5	Fig. 2j
		Ep400ΔPNS	66 ± 10	Fig. 2j
	P21	control	8 ± 2	Fig. 2j
		Ep400ΔPNS	65 ± 6	Fig. 2j
	2 months	control	3 ± 0	Fig. 2j
		Ep400ΔPNS	6 ± 2	Fig. 2j
Iba1	P0	control	1047 ± 58	Fig. 2k
		control	970 ± 24	Fig. 5u
		Ep400ΔPNS	927 ± 50	Fig. 2k
		Ep400ΔPNS	991 ± 36	Fig. 5u
		dko	978 ± 30	Fig. 5u
	P9	control	593 ± 49	Fig. 2k
		control	460 ± 24	Fig. 5u
		Ep400ΔPNS	1077 ± 124	Fig. 2k
		Ep400ΔPNS	1015 ± 7	Fig. 5u
		dko	770 ± 76	Fig. 5u
	P15	control	260 ± 70	Fig. 2k
		Ep400ΔPNS	1220 ± 105	Fig. 2k
	P21	control	103 ± 35	Fig. 2k
		control	27 ± 6	Fig. 5u
		Ep400ΔPNS	617 ± 34	Fig. 2k
		Ep400ΔPNS	337 ± 55	Fig. 5u
		dko	161 ± 20	Fig. 5u
	2 months	control	120 ± 17	Fig. 2k
		control	131 ± 4	Fig. 5u
		Ep400ΔPNS	340 ± 72	Fig. 2k
		Ep400ΔPNS	340 ± 19	Fig. 5u
		dko	332 ± 10	Fig. 5u
Ki67	P0	control	2027 ± 127	Suppl. Fig. 5f
		Ep400ΔPNS	2163 ± 253	Suppl. Fig. 5f
	P9	control	357 ± 69	Suppl. Fig. 5f
		Ep400ΔPNS	963 ± 171	Suppl. Fig. 5f
	P15	control	37 ± 9	Suppl. Fig. 5f
		Ep400ΔPNS	503 ± 70	Suppl. Fig. 5f

	P21	control	17± 7	Suppl. Fig. 5f
		Ep400ΔPNS	333 ± 62	Suppl. Fig. 5f
	2 months	control	13 ± 3	Suppl. Fig. 5f
		Ep400ΔPNS	77 ± 23	Suppl. Fig. 5f
Cleaved caspase 3	P0	control	507 ± 72	Suppl. Fig. 5h
		Ep400ΔPNS	537 ± 90	Suppl. Fig. 5h
	P9	control	19 ± 3	Suppl. Fig. 5h
		Ep400ΔPNS	90 ± 7	Suppl. Fig. 5h
	P15	control	24 ± 2	Suppl. Fig. 5h
		Ep400ΔPNS	127 ± 14	Suppl. Fig. 5h
	P21	control	18 ± 1	Suppl. Fig. 5h
		Ep400ΔPNS	82 ± 3	Suppl. Fig. 5h
	2 months	control	11 ± 2	Suppl. Fig. 5h
		Ep400ΔPNS	16 ± 2	Suppl. Fig. 5h

Supplementary Table 6: Determination of transcript levels in sciatic nerves of Ep400ΔPNS mice relative to control

Gene	Primer	Age	Mean value ± SEM	Figure
Ep400	5’-GCAGATGCAGACATCTCAGC-3’<br>5’-GTTGGTAGCAGAAGAGGGTC-3’	P9	0.57 ± 0.04	Fig. 2l
		P21	0.47 ± 0.08	Fig. 2l
		2 months	0.57 ± 0.06	Fig. 2l
ErbB3	5’-TGTGGACAGCAAAACCTCAG-3’<br>5’-CCATCCTGGGCTACACAGTT-3’	P9	1.00 ± 0.09	Fig. 2l
		P21	1.08 ± 0.13	Fig. 2l
		2 months	1.66 ± 0.25	Fig. 2l
S100b	5'- GACTCCAGCAGCAAAGGTGAC- 3'<br>5'-CATCTTCGTCCAGCGTCTCCA-3'	P9	0.62 ± 0.16	Fig. 2l
		P21	0.31 ± 0.11	Fig. 2l
		2 months	0.66 ± 0.15	Fig. 2l
Fabp7	5‘-TTTGTCTTCGGTGTGGAGGT-3‘<br>5’-TGGTTTGCATTCCAAAGAAAG-3’	P9	0.45 ± 0.16	Fig. 2l
		P21	0.67 ± 0.33	Fig. 2l
		2 months	0.87 ± 0.15	Fig. 2l
Cdh19	5’-GGGAGAATTTCCTCCTCTGG-3’<br>5’-CGGAAAATTTCTCAATGACCA-3’	P9	1.24 ± 0.12	Fig. 2l
		P21	0.83 ± 0.29	Fig. 2l
		2 months	1.67 ± 0.15	Fig. 2l
L1Cam	5’-CTCCTCCCATTGCTATTCCA-3’<br>5’-AGCCAGTGCCTGTAGGAAGA-3’	P9	0.72 ± 0.12	Fig. 2l
		P21	0.89 ± 0.28	Fig. 2l
		2 months	1.39 ± 0.18	Fig. 2l
Dhh	5'-CTTGGACATCACCACGTCTG-3'<br>5'-GTAGTTCCCTCAGCCCCTTC-3'	P9	1.65 ± 0.27	Fig. 2l
		P21	2.33 ± 0.09	Fig. 2l
		2 months	5.07 ± 0.48	Fig. 2l
Nfasc	5‘-GACAGCCTGGTGGACTATGG-3‘<br>5’-TCTGCAGTTGCTTCATCTCC-3’	P9	0.15 ± 0.05	Fig. 2l
		P21	0.19 ± 0.05	Fig. 2l
		2 months	0.37 ± 0.15	Fig. 2l
Ank2	5’-GCTTCAGGGGTGGTGTTCTA-3’<br>5’-TACAGTGCAGTGGCCAGAAG-3’	P9	0.62 ± 0.14	Fig. 2l
		P21	0.50 ± 0.16	Fig. 2l
		2 months	0.37 ± 0.03	Fig. 2l
Sox10	5’-ACAGCAGCAGGAAGGCTTCT-3’<br>5’-TGTCCTCAGTGCGTCCTTAG-3’	P9	0.86 ± 0.03	Fig. 2m
		P21	0.56 ± 0.20	Fig. 2m
		2 months	1.21 ± 0.21	Fig. 2m
Oct6	5’-GTTCTCGCAGACCACCATCT-3’<br>5’-GGCTTGGGACACTTGAGAAA-3’	P9	0.67 ± 0.11	Fig. 2m
		P21	1.49 ± 0.32	Fig. 2m
		2 months	3.83 ± 0.36	Fig. 2m
Krox20	5’-AGGCCCCTTTGACCAGATGA-3’<br>5’-AAGATGCCCGCACTCACAAT-3’	P9	0.45 ± 0.05	Fig. 2m
		P21	0.23 ± 0.03	Fig. 2m
		2 months	0.41 ± 0.08	Fig. 2m
Cx32	5’-CTGCTCTACCCCGGCTATGC-3’	P9	0.19 ± 0.03	Fig. 2m

	5’-CAGGCTGAGCATCGGTCGCTCTT-3’	P21	0.20 ± 0.01	Fig. 2m
		2 months	0.71 ± 0.14	Fig. 2m
Mbp	5’-CACACACGAGAACTACCCA- 3’<br>5’-CCAAGTTCACCCCTACTCCA-3’	P9	0.24 ± 0.04	Fig. 2m
		P21	0.14 ± 0.01	Fig. 2m
		2 months	0.24 ± 0.01	Fig. 2m
Mpz	5’-CTGGTCCAGTGAATGGGTCT-3’<br>5’-CATGTGAAAGTGCCGTTGTC-3’	P9	0.25 ± 0.07	Fig. 2m
		P21	0.10 ± 0.03	Fig. 2m
		2 months	0.36 ± 0.04	Fig. 2m
Pmp22	5’-CTCAAAGCCTTCGTCAGTCC-3’<br>5’-TTGGTGGCCAATACAAGTCA-3’	P9	0.28 ± 0.07	Fig. 2m
		P21	0.16 ± 0.03	Fig. 2m
		2 months	0.36 ± 0.06	Fig. 2m
Periaxin	5’-TAGTGGGTGAGGGCATCTTC-3’<br>5’-TGGTGACGTGAGTTCCACAT-3’	P9	0.36 ± 0.11	Fig. 2m
		P21	0.25 ± 0.10	Fig. 2m
		2 months	0. 65 ± 0.14	Fig. 2m
Tfap2a	5’-GCTCCACCTCGAAGTACAAG-3’<br>5’-CCTGCTGGCAGATTCAATCC-3’	P9	18.36 ± 2.43	Fig. 4i
		P21	65.82 ± 13.73	Fig. 4i
		2 months	153.9 ± 11.66	Fig. 4i
Pou3f3	5’-TCCAGAACAGGCTAGGGTTG-3’<br>5’-GGTGGGCATATTTTCACTGG-3’	P9	57.42 ± 18.64	Fig. 4i
		P21	1.40 ± 0.09	Fig. 4i
		2 months	3.30 ± 0.54	Fig. 4i
Pax3	5’-GGGAACTGGAGGCATGTTTA-3’<br>5’-GTTTTCCGTCCCAGCAATTA-3’	P9	44.64 ± 12.09	Fig. 4i
		P21	4.36 ± 0.72	Fig. 4i
		2 months	6.58 ± 1.10	Fig. 4i
Sox1	5’-GAGGATGGGTTGTGCTCAGT-3’<br>5’-TGGGATAAGACCTGGGTGAG-3’	P9	61.88 ± 8.68	Fig. 4i
		P21	2.26 ± 0.31	Fig. 4i
		2 months	7.49 ± 1.28	Fig. 4i
Sox2	5’-CACAACTCGGAGATCAGCAA-3’<br>5’-CTCCGGGAAGCGTGTACTTA3’	P9	1.30 ± 0.03	Fig. 4i
		P21	1.42 ± 0.05	Fig. 4i
		2 months	2.55 ± 0.22	Fig. 4i
Sox3	5’-CGTAACTGTCGGGGTTTTGT-3’<br>5’-AACCTAGGAATCCGGGAAGA-3’	P9	2.33 ± 0.26	Fig. 4i
		P21	2.67 ± 0.29	Fig. 4i
		2 months	8.88 ± 0.56	Fig. 4i

Supplementary Table 7: Percentage of Krox20-positive SCs that express Sox2

Marker	Age	Genotype	Mean value ± SEM	Figure
Sox2 + Krox20	P21	control	0.28 ± 0.28	Fig. 4h
		Ep400ΔPNS	5.68 ± 0.68	Fig. 4h

Supplementary Table 8: Effect of transcription factors on SC-specific regulatory regions in luciferase reporter gene assays

Reporter	Effector	Mean value ± SEM	Figure
Krox20 MSE-luc	-	1.00 ± 0.00	Fig. 4k
	Sox10	45.17 ± 0.80	Fig. 4k
	Sox10 + Pax3	1.26 ± 0.15	Fig. 4k
	Sox10 + Sox2	11.42 ± 2.67	Fig. 4k
	Sox10 + Sox3	4.75 ± 1.21	Fig. 4k
	Sox10 + Tfap2a	9.84 ± 3.94	Fig. 4k
Pmp22-luc	-	1.00 ± 0.00	Fig. 4l
	Krox20	19.67 ± 1.59	Fig. 4l
	Krox20 + Pax3	3.77 ± 0.20	Fig. 4l
	Krox20 + Sox2	23.67 ± 1.90	Fig. 4l
	Krox20 + Sox3	4.47 ± 0.35	Fig. 4l
	Krox20 + Tfap2a	26.17 ± 1.93	Fig. 4l
Periaxin-luc	-	1.00 ± 0.00	Fig. 4m
	Krox20	66.17 ± 10.88	Fig. 4m
	Krox20 + Pax3	10.93 ± 2.11	Fig. 4m
Mag-luc	-	1.00 ± 0.00	Fig. 4m
	Krox20	27.20 ± 2.43	Fig. 4m
	Krox20 + Pax3	7.37 ± 1.52	Fig. 4m
Connexin32-luc	-	1.00 ± 0.00	Fig. 4m
	Krox20	8.20 ± 0.46	Fig. 4m
	Krox20 + Pax3	4.77 ± 0.35	Fig. 4m
Mpz-luc	-	1.00 ± 0.00	Fig. 4m
	Krox20	10.27 ± 1.13	Fig. 4m
	Krox20 + Pax3	2.73 ± 0.09	Fig. 4m

Supplementary Table 9: Quantification of myelin debris, myelin outfoldings and Remak bundles in sciatic nerves of control and mutant mice

Structure	Age	Genotype	Mean value ± SEM	Figure
Myelin debris	P21	control	13 ± 7	Fig.5o, Suppl. Fig.3s
		Ep400ΔPNS	434 ± 26	Fig.5o, Suppl. Fig.3s
		dko	220 ± 31	Fig.5o
	2 months	control	4 ± 4	Suppl. Fig.3t
		Ep400ΔPNS	301 ± 38	Suppl. Fig.3t
Myelin outfoldings	P21	control	52 ± 43	Fig.5p, Suppl. Fig.3u
		Ep400ΔPNS	656 ± 52	Fig.5p, Suppl. Fig.3u
		dko	324 ± 86	Fig.5p
	2 months	control	42 ± 15	Suppl. Fig.3v
		Ep400ΔPNS	1445 ± 92	Suppl. Fig.3v
Remak bundles	P21	control	889 ± 2	Suppl. Figs.3w & 8g
		Ep400ΔPNS	697 ± 9	Suppl. Figs.3w & 8g
		dko	738 ± 19	Suppl. Fig.8g
	2 months	control	828 ± 50	Suppl. Figs.3x
		Ep400ΔPNS	645 ± 32	Suppl. Figs.3x

Supplementary Table 10: Number of Ep400-positive SCs in control and mutant mice

Age	Genotype	Mean value ± SEM	Figure
E12.5	control	95.1 ± 0.4	Suppl. Fig.2a
	Ep400ΔNC	5.7 ± 0.6	Suppl. Fig.2a
E14.5	control	93.4 ± 1.1	Suppl. Fig.2a
	Ep400ΔNC	5.8 ± 1.0	Suppl. Fig.2a
P0	control	99.3 ± 0.2	Suppl. Fig. 3a
	Ep400ΔPNS	1.7 ± 0.5	Suppl. Fig. 3a
P21	control	99.8 ± 0.2	Suppl. Fig. 3a
	Ep400ΔPNS	6.3 ± 1.7	Suppl. Fig. 3a

Supplementary Table 11: Relative number of cycling, proliferating and just differentiating SCs in control and mutant mice

Feature	Genotype	Mean value ± SEM	Figure
Ki67+	control	0.44 ± 0.04	Suppl. Fig.5a
	Ep400ΔNC	0.40 ± 0.04	Suppl. Fig.5a
Ki67+, BrdU+	control	0.15 ± 0.02	Suppl. Fig.5b
	Ep400ΔNC	0.14 ± 0.05	Suppl. Fig.5b
Ki67+, BrdU-	control	0.33 ± 0.05	Suppl. Fig.5c
	Ep400ΔPNS	0.24 ± 0.01	Suppl. Fig.5c
Ki67-, EdU+	control	0.51 ± 0.03	Suppl. Fig.5d
	Ep400ΔPNS	0.54 ± 0.05	Suppl. Fig.5d

Supplementary Table 12: Primers used to amplify specific regions from the mouse *Tfap2a* and *Pax3* genomic loci

Gene	Region	Position (mm10)	Primer
Tfap2a	1	chr13, 40739401 - 40739420	5'-ACGATAGTAGCGCCAGGAGA-3'
		chr13, 40739315 - 40739334	5'-AGTTGCGGCAGTCACTTTCT-3'
	2	chr13, 40735201 - 40735220	5'-AGTGGGAAAACTCAGCCAGA-3'
		chr13, 40735103 - 40735122	5'-GGACCCTTCCTCCCTTACTG-3'
	3	chr13, 40732357 - 40732376	5'-GCCTTTCTCCTCTCCGTCTT-3'
		chr13, 40732231 - 40732250	5'-TCTGGTCCTACATGGGGTTC-3'
	4	chr13, 40731944 - 40731963	5'-CATGAGAGAGGGGTTGGAGA-3'
		chr13, 40731856 - 40731875	5'-GCTATCCCGCTGATACCAAA-3'
	5	chr13, 40731514 - 40731532	5'-ACCAACCGAGATCGGAGAA-3'
		chr13, 40731434 - 40731453	5'-GCTTCGCTAACCCTTGTCCT-3'
	6	chr13, 40730811 - 40730830	5'-GTCGCTCGCTTTTTGTCTCT-3'
		chr13, 40730689 - 40730708	5'-AGGGAAGAATGCCTGGAAAT-3'
	7	chr13, 40730407 - 40730426	5'-TTGTTGTTGGTGGTGTGGTT-3'
		chr13, 40730295 - 40730314	5'-CCGAACTTGTACCACCGAGT-3'
	8	chr13, 40729453 - 40729472	5'-GGTGCGGCAGAATCTTTCTA-3'
		chr13, 40729365 - 40729384	5'-CGCTTCCTCTGTTTGTAGGC-3'
	9	chr13, 40726704 - 40726723	5'-CAGGAAGCCTTGTGCTCTTC-3'
		chr13, 40726609 - 40726628	5'-CTGTGGCAGCAATGAGAAAA-3'
	10	chr13, 40724516 - 40724535	5'-TGCATGGGTTTATCATCTGC-3'
		chr13, 40724432 - 40724451	5'-CCTGAAGTACCTGCCACCAT-3'
Pax3	1	chr1, 78200684 - 78200703	5'-AGATCCGCATCTTGCTCCTA-3'
		chr1, 78200595 - 78200614	5'-CCACACCTCCGCAGTTATTT-3'
	2	chr1, 78199415 - 78199434	5'-CTCTCAGTGCCCTCAGAACC-3'
		chr1, 78199287 - 78199306	5'-TACTCAGAGGCAGCCAGGAT-3'
	3	chr1, 78197497 - 78197516	5'-GAGGAAATAAGGGGCGGATA-3'
		chr1, 78197399 - 78197418	5'-GGTGTCCCTGTCCCTCTACA-3'
	4	chr1, 78197229 - 78197248	5'-GTAGCTTCTGTCGCCCAATC-3'
		chr1, 78197121 - 78197140	5'-ATCCGGACTAGGGAGCATTT-3'
	5	chr1, 78196977 - 78196996	5'-TCTGCTAGACTCGCACCAAA-3'
		chr1, 78196900 - 78196919	5'-GGGGCGCTAATAGATCCAAG-3'
	6	chr1, 78196541 - 78196560	5'-TCCCGTGGTACTTCTCAACC-3'
		chr1, 78196455 - 78196474	5'-GAGCTTAGCACTCCGTTTGC-3'
	7	chr1, 78196120 - 78196139	5'-TCGGTTTGCTTAAAGGTGCT-3'
		chr1, 78196038 - 78196057	5'-AACCCCAGTCTCCTGCTTTT-3'
	8	chr1, 78194974 - 78194993	5'-GATTTTAATGCCTGGGAGCA-3'
		chr1, 78194906 - 78194925	5'-CAGTGCAGTGAGTGGGAAGA-3'
	9	chr1, 78194172 - 78194191	5'-GTTTCGTTTTTGTGCGGATT-3'
		chr1, 78194076 - 78194095	5'-GATGCTCGGGAACCAAATTA-3'

Supplementary Table 13: H2A.Z and H3K27ac occupancy at specific *Tfap2a* and *Pax3* genomic regions in sciatic nerves of control and Ep400ΔPNS mice

Gene & Region	Age	Histone	Enrichment in control (mean ± SEM)	Enrichment in Ep400ΔPNS (mean ± SEM)	Figure
Tfap2a 1	P9	H2A.Z	1.06 ± 0.08	0.96 ± 0.29	Suppl. Fig. 7e
2		H2A.Z	1.00 ± 0.07	0.64 ± 0.22	Suppl. Fig. 7e
3		H2A.Z	0.86 ± 0.05	1.11 ± 0.44	Suppl. Fig. 7e
4		H2A.Z	0.90 ± 0.13	6.73 ± 0.24	Suppl. Fig. 7e
5		H2A.Z	2,71 ± 0.47	18.43 ± 1.12	Suppl. Fig. 7e
6		H2A.Z	3.65 ± 0.58	4.44 ± 0.37	Suppl. Fig. 7e
7		H2A.Z	1.88 ± 0.43	5.45 ± 1.22	Suppl. Fig. 7e
8		H2A.Z	1.02 ± 0.04	3.69 ± 0.71	Suppl. Fig. 7e
9		H2A.Z	1.11 ± 0.27	1.03 ± 0.13	Suppl. Fig. 7e
10		H2A.Z	0.95 ± 0.11	1.12 ± 0.01	Suppl. Fig. 7e
Tfap2a 1		H3K27ac	0.89 ± 0.21	1.05 ± 0.14	Suppl. Fig. 7h
2		H3K27ac	1.12 ± 0.09	0.92 ± 0.09	Suppl. Fig. 7h
3		H3K27ac	0.82 ± 0.17	2.29 ± 0.44	Suppl. Fig. 7h
4		H3K27ac	1.12 ± 0.15	8.31 ± 2.51	Suppl. Fig. 7h
5		H3K27ac	0.78 ± 0.11	29.21 ± 1.91	Suppl. Fig. 7h
6		H3K27ac	0.93 ± 0.09	3.22 ± 0.57	Suppl. Fig. 7h
7		H3K27ac	0.93 ± 0.32	5.20 ± 1.47	Suppl. Fig. 7h
8		H3K27ac	0.70 ± 0.24	0.91 ± 0.19	Suppl. Fig. 7h
9		H3K27ac	0.86 ± 0.26	0.91 ± 0.28	Suppl. Fig. 7h
10		H3K27ac	0.92 ± 0.23	1.11 ± 0.16	Suppl. Fig. 7h
Pax3 1		H2A.Z	1.00 ± 0.27	0.92 ± 0.43	Suppl. Fig. 7l
2		H2A.Z	1.05 ± 0.23	0.84 ± 0.42	Suppl. Fig. 7l
3		H2A.Z	1.75 ± 0.67	6.79 ± 0.90	Suppl. Fig. 7l
4		H2A.Z	3.69 ± 0.41	4.82 ± 0.55	Suppl. Fig. 7l
5		H2A.Z	1.04 ± 0.23	5.95 ± 1.53	Suppl. Fig. 7l
6		H2A.Z	0.75 ± 0.06	3.06 ± 0.26	Suppl. Fig. 7l
7		H2A.Z	1.11 ± 0.17	1.08 ± 0.34	Suppl. Fig. 7l
8		H2A.Z	1.11 ± 0.49	1.03 ± 0.12	Suppl. Fig. 7l
9		H2A.Z	0.91 ± 0.14	1.32 ± 0.23	Suppl. Fig. 7l
Pax3 1		H3K27ac	0.74 ± 0.02	0.65 ± 0.24	Suppl. Fig. 7o
2		H3K27ac	0.81 ± 0.26	0.76 ± 0.22	Suppl. Fig. 7o
3		H3K27ac	0.73 ± 0.19	1.57 ± 0.64	Suppl. Fig. 7o
4		H3K27ac	1.06 ± 0.23	2.83 ± 0.22	Suppl. Fig. 7o
5		H3K27ac	0.79 ± 0.02	3.81 ± 0.86	Suppl. Fig. 7o
6		H3K27ac	0.58 ± 0.10	0.74 ± 0.27	Suppl. Fig. 7o
7		H3K27ac	0.67 ± 0.07	0.84 ± 0.35	Suppl. Fig. 7o

8		H3K27ac	0.73 ± 0.22	0.57 ± 0.06	Suppl. Fig. 7o
9		H3K27ac	0.79 ± 0.41	1.12 ± 0.32	Suppl. Fig. 7o
Tfap2a 1	P21	H2A.Z	3.38 ± 1.04	4.76 ± 1.35	Suppl. Fig. 7f
2		H2A.Z	4.70 ± 1.25	6.69 ± 1.55	Suppl. Fig. 7f
3		H2A.Z	8.50 ± 2.16	6.25 ± 2.21	Suppl. Fig. 7f
4		H2A.Z	5.78 ± 1.30	13.10 ± 0.89	Suppl. Fig. 7f
5		H2A.Z	26.79 ± 5.21	14.25 ± 1.14	Suppl. Fig. 7f
6		H2A.Z	17.03 ± 2.22	36.95 ± 7.36	Suppl. Fig. 7f
7		H2A.Z	14.11 ± 5.43	15.22 ± 4.47	Suppl. Fig. 7f
8		H2A.Z	3.98 ± 0.44	8.89 ± 2.44	Suppl. Fig. 7f
9		H2A.Z	1.56 ± 0.71	2.45 ± 0.48	Suppl. Fig. 7f
10		H2A.Z	6.31 ± 1.53	4.41 ± 1.19	Suppl. Fig. 7f
Tfap2a 1		H3K27ac	1.22 ± 0.15	1.76 ± 0.27	Suppl. Fig. 7i
2		H3K27ac	1.81 ± 0.30	2.12 ± 0.55	Suppl. Fig. 7i
3		H3K27ac	2.44 ± 0.29	6.62 ± 0.70	Suppl. Fig. 7i
4		H3K27ac	0.86 ± 0.15	11.98 ± 3.04	Suppl. Fig. 7i
5		H3K27ac	3.79 ± 1.49	12.22 ± 0.13	Suppl. Fig. 7i
6		H3K27ac	2.44 ± 0.30	14.57 ± 2.93	Suppl. Fig. 7i
7		H3K27ac	2.16 ± 0.95	15.99 ± 3.57	Suppl. Fig. 7i
8		H3K27ac	1.42 ± 0.48	5.02 ± 0.34	Suppl. Fig. 7i
9		H3K27ac	0.96 ± 0.57	3.37 ± 0.40	Suppl. Fig. 7i
10		H3K27ac	2.67 ± 0.66	5.31 ± 1.59	Suppl. Fig. 7i
Pax3 1		H2A.Z	1.44 ± 0.28	2.85 ± 0.36	Suppl. Fig. 7m
2		H2A.Z	1.96 ± 1.10	3.75 ± 0.89	Suppl. Fig. 7m
3		H2A.Z	11.06 ± 2.05	9.44 ± 2.63	Suppl. Fig. 7m
4		H2A.Z	11.84 ± 0.52	20.42 ± 4.74	Suppl. Fig. 7m
5		H2A.Z	4.56 ± 0.71	29.90 ± 5.09	Suppl. Fig. 7m
6		H2A.Z	6.82 ± 0.45	21.82 ± 5.75	Suppl. Fig. 7m
7		H2A.Z	3.36 ± 1.02	5.59 ± 0.05	Suppl. Fig. 7m
8		H2A.Z	1.66 ± 0.83	0.56 ± 0.00	Suppl. Fig. 7m
9		H2A.Z	3.54 ± 0.48	3.19 ± 1.36	Suppl. Fig. 7m
Pax3 1		H3K27ac	1.26 ± 0.47	1.07 ± 0.32	Suppl. Fig. 7p
2		H3K27ac	0.73 ± 0.16	1.69 ± 0.88	Suppl. Fig. 7p
3		H3K27ac	1.47 ± 0.09	1.63 ± 0.14	Suppl. Fig. 7p
4		H3K27ac	1.54 ± 0.20	9.64 ±2.42	Suppl. Fig. 7p
5		H3K27ac	2.61 ± 1.62	6.60 ± 0.82	Suppl. Fig. 7p
6		H3K27ac	2.69 ± 0.84	4.66 ± 0.57	Suppl. Fig. 7p
7		H3K27ac	1.19 ± 0.04	2.72 ± 0.68	Suppl. Fig. 7p
8		H3K27ac	1.86 ± 0.62	0.28 ± 0.00	Suppl. Fig. 7p
9		H3K27ac	1.327 ± 0.33	3.34 ± 0.67	Suppl. Fig. 7p
Tfap2a 1	2months	H2A.Z	1.987 ± 0.34	2.94 ± 0.49	Suppl. Fig. 7g
2		H2A.Z	1.863 ± 0.45	1.31 ± 0.15	Suppl. Fig. 7g
3		H2A.Z	2.377 ± 0.24	2.12 ± 0.36	Suppl. Fig. 7g

4		H2A.Z	2.570 ± 0.70	6.74 ± 0.54	Suppl. Fig. 7g
5		H2A.Z	10.033 ± 2.76	22.18 ± 3.04	Suppl. Fig. 7g
6		H2A.Z	8.193 ± 0.76	15.23 ± 2.61	Suppl. Fig. 7g
7		H2A.Z	4.813 ± 1.15	6.56 ± 0.82	Suppl. Fig. 7g
8		H2A.Z	2.163 ± 0.24	4.14 ± 0.16	Suppl. Fig. 7g
9		H2A.Z	1.033 ± 0.36	2.31 ± 0.17	Suppl. Fig. 7g
10		H2A.Z	1.627 ± 0.26	2.12 ± 0.45	Suppl. Fig. 7g
Tfap2a 1		H3K27ac	2.653 ± 0.56	1.52 ± 0.28	Suppl. Fig. 7j
2		H3K27ac	2.453 ± 0.84	2.52 ± 0.44	Suppl. Fig. 7j
3		H3K27ac	2.003 ± 0.54	4.19 ± 0.77	Suppl. Fig. 7j
4		H3K27ac	2.223 ± 0.44	16.64 ± 0.29	Suppl. Fig. 7j
5		H3K27ac	4.747 ± 1.44	68.71 ± 4.79	Suppl. Fig. 7j
6		H3K27ac	3.630 ± 1.05	12.47 ± 1.66	Suppl. Fig. 7j
7		H3K27ac	2.420 ± 0.90	9.91 ± 1.44	Suppl. Fig. 7j
8		H3K27ac	2.757 ± 0.23	3.25 ±0.40	Suppl. Fig. 7j
9		H3K27ac	1.333 ± 0.57	3.01 ± 0.50	Suppl. Fig. 7j
10		H3K27ac	2.133 ± 0.79	1.11 ± 0.43	Suppl. Fig. 7j
Pax3 1		H2A.Z	1.000 ± 0.27	2.27 ± 0.55	Suppl. Fig. 7n
2		H2A.Z	1.497 ± 0.21	3.55 ± 0.50	Suppl. Fig. 7n
3		H2A.Z	19.180 ± 1.87	10.68 ± 1.94	Suppl. Fig. 7n
4		H2A.Z	5.480 ± 1.01	8.27 ± 0.76	Suppl. Fig. 7n
5		H2A.Z	2.343 ± 0.44	22.64 ± 3.42	Suppl. Fig. 7n
6		H2A.Z	4.353 ± 0.36	10.42 ± 2.36	Suppl. Fig. 7n
7		H2A.Z	2.717 ± 0.79	3.74 ± 0.39	Suppl. Fig. 7n
8		H2A.Z	1.470 ± 0.49	3.48 ± 1.54	Suppl. Fig. 7n
9		H2A.Z	2.773 ± 1.25	1.98 ± 0.38	Suppl. Fig. 7n
Pax3 1		H3K27ac	1.110 ± 0.29	0.85 ± 0.17	Suppl. Fig. 7q
2		H3K27ac	2.203 ± 0.25	1.16 ± 0.01	Suppl. Fig. 7q
3		H3K27ac	2.797 ± 0.70	2.10 ± 0.12	Suppl. Fig. 7q
4		H3K27ac	1.867 ± 0.21	6.83 ± 1.47	Suppl. Fig. 7q
5		H3K27ac	0.927 ± 0.42	7.39 ± 0.72	Suppl. Fig. 7q
6		H3K27ac	1.757 ± 0.28	3.16 ± 0.35	Suppl. Fig. 7q
7		H3K27ac	1.030 ± 0.33	2.65 ± 0.41	Suppl. Fig. 7q
8		H3K27ac	1.950 ± 0.17	2.22 ± 0.26	Suppl. Fig. 7q
9		H3K27ac	1.283 ± 0.32	1.05 ± 0.19	Suppl. Fig. 7q
