# Supplementary material for: Ep400 deficiency in Schwann cells causes persistent expression of early developmental regulators and peripheral neuropathy
Source: Nat Commun. 2019 May 29;10:2361. doi: 10.1038/s41467-019-10287-w (PMC6541636; doi:10.1038/s41467-019-10287-w)
Supplement: Supplementary file 3 — Reporting Summary [file 41467_2019_10287_MOESM3_ESM.pdf]

nature research

Corresponding author(s): Michael Wegner

Last updated by author(s): April 22, 2019

Reporting Summary

Nature Research wishes to improve the reproducibility of the work that we publish. This form provides structure for consistency and transparency in reporting. For further information on Nature Research policies, see Authors & Referees and the Editorial Policy Checklist.

Statistics

For all statistical analyses, confirm that the following items are present in the figure legend, table legend, main text, or Methods section.

n/a	Confirmed	
☐	☒	The exact sample size (n) for each experimental group/condition, given as a discrete number and unit of measurement
☐	☒	A statement on whether measurements were taken from distinct samples or whether the same sample was measured repeatedly
☐	☒	The statistical test(s) used AND whether they are one- or two-sided<br>*Only common tests should be described solely by name; describe more complex techniques in the Methods section.*
☐	☒	A description of all covariates tested
☐	☒	A description of any assumptions or corrections, such as tests of normality and adjustment for multiple comparisons
☐	☒	A full description of the statistical parameters including central tendency (e.g. means) or other basic estimates (e.g. regression coefficient) AND variation (e.g. standard deviation) or associated estimates of uncertainty (e.g. confidence intervals)
☒	☐	For null hypothesis testing, the test statistic (e.g. F, t, r) with confidence intervals, effect sizes, degrees of freedom and P value noted<br>*Give P values as exact values whenever suitable.*
☒	☐	For Bayesian analysis, information on the choice of priors and Markov chain Monte Carlo settings
☒	☐	For hierarchical and complex designs, identification of the appropriate level for tests and full reporting of outcomes
☒	☐	Estimates of effect sizes (e.g. Cohen's d, Pearson's r), indicating how they were calculated

Our web collection on statistics for biologists contains articles on many of the points above.

Software and code

Policy information about availability of computer code

Data collection	For RNA-Seq:<br>- sequence alignment: STAR (version 2.5.1.b)<br>For ChIP-Seq:<br>- read mapping: Bowtie (version 2.3.4.2)<br>- removing PCR duplicates: RmDup (version 2.0.1)<br>- convertion to BAM format: SAMtools (version 1.1.2)
Data analysis	For RNA-Seq:<br>- differential expression analysis: DESeq2 package (version 1.10.1)<br>For ChIP-Seq:<br>- BamCoverage tool within the deeptools software (version 3.0.2.0)<br>- peak calling Model-based Analysis of ChIP-Seq (MACS2) (version 2.1.1.20160309.0)<br>- visualization: Integrated Genome Viewer (IGV; version 2.3.98)<br>- heatmaps and plotprofiles: deeptools software computeMatrix scale region mode (version 2.5.0.0)<br>- differential binding analysis: DiffBind (version 2.6.6)<br>- intersection with genes: intersect (version 1.0.0)<br>- connection of peak lists: join (version 1.0.0)

For manuscripts utilizing custom algorithms or software that are central to the research but not yet described in published literature, software must be made available to editors/reviewers. We strongly encourage code deposition in a community repository (e.g. GitHub). See the Nature Research guidelines for submitting code & software for further information.

Data

Policy information about availability of data

All manuscripts must include a data availability statement. This statement should provide the following information, where applicable:

- Accession codes, unique identifiers, or web links for publicly available datasets
- A list of figures that have associated raw data
- A description of any restrictions on data availability

All data generated or analyzed during this study are included in this published article and its supplementary information files or are available in the GEO database under accession numbers GSE119132 (RNA-Seq) and GSE119194 (ChIP-Seq).

Field-specific reporting

Please select the one below that is the best fit for your research. If you are not sure, read the appropriate sections before making your selection.

☒ Life sciences ☐ Behavioural & social sciences ☐ Ecological, evolutionary & environmental sciences

For a reference copy of the document with all sections, see nature.com/documents/nr-reporting-summary-flat.pdf

Life sciences study design

All studies must disclose on these points even when the disclosure is negative.

Sample size	Sample size was n ≥ 3 for all molecular biology experiments and experiments with mice as common for this kind of study except for ChIP-Seq studies with anti-H3K27ac antibodies (n =2).
Data exclusions	No data were excluded from the analysis.
Replication	Part of the experiments were carried out in different laboratories, others were repeated after more than a year with consistent results.
Randomization	Randomization was not possible.
Blinding	Investigators were not blinded in animal experiments, but in the following quantifications

Reporting for specific materials, systems and methods

We require information from authors about some types of materials, experimental systems and methods used in many studies. Here, indicate whether each material, system or method listed is relevant to your study. If you are not sure if a list item applies to your research, read the appropriate section before selecting a response.

Materials & experimental systems

n/a	Involved in the study	
☐	☒	Antibodies
☐	☒	Eukaryotic cell lines
☒	☐	Palaeontology
☐	☒	Animals and other organisms
☒	☐	Human research participants
☒	☐	Clinical data

Methods

n/a	Involved in the study	
☐	☒	ChIP-seq
☒	☐	Flow cytometry
☒	☐	MRI-based neuroimaging

Antibodies

Antibodies used

rat anti-MBP monoclonal (Bio-Rad, #MCA409S, Lot #210610), rat anti-BrdU monoclonal (Abcam, #ab6326, Lot#GR3173637-7), , rabbit anti-Caspr antiserum (Abcam, #AB34151, Lot#GR86230), guinea pig anti-Sox10 antiserum (homemade, validated on control and knockout mouse tissue) [Maka, 2005 #1189], rabbit anti-Ep400 antiserum (homemade, generated against a bacterially expressed and purified peptide corresponding to amino acids 507-661 of mouse Ep400 according to accession number NP_083613, validated on control and knockout mouse tissue), guinea pig anti-Krox20 antiserum (homemade, generated against a bacterially expressed and purified peptide corresponding to amino acids 28-166 of mouse Krox20 according to accession number NM_010118.3, validated on control and knockout mouse tissue), rabbit anti-Sox2 antiserum (homemade, generated against a bacterially expressed and purified peptide corresponding to amino acids 10-38 of mouse Sox2 according to accession number NM_011443, validated on control and knockout mouse tissue), rabbit anti-Oct6 antiserum (homemade, generated against baculovirus-expressed and purified full length mouse Oct6 according to accession number NM_011141.2, validated on control and knockout mouse tissue), rabbit anti-Nav1.6 antiserum (Alomone Labs, #ASC-009, Lot#ASC009AN2425, 1:50 dilution), rabbit anti-Iba1 antiserum (Wako, #019-19741, Lot#SAE6921), rabbit anti-cleaved caspase 3 antiserum (Cell Signaling Technology, #9661, Lot#0043), rabbit anti-Ki67 antiserum (Thermo Fisher Scientific, #RM-9106, Lot#9106S906D), rabbit

	anti-CD3 antiserum (Abcam, #ab5690, Lot#665620), rabbit anti-Desmin antiserum (Abcam, #ab15200), rabbit anti-fibronectin antiserum (Abcam, #ab2413, Lot#GR3235936-2), mouse anti-alpha smooth muscle actin antiserum (Sigma, #A5228), rabbit antiserum against H3K27ac (Diagenode, #C15410196, Lot#A1723-0041D), rabbit antiserum against H2A.Z (Diagenode, #C15410201, Lot#A2039P).
Validation	All commercially available antibodies are regularly used by the community in the assays performed in this study and have been independently validated numerous times, among others by the manufacturer as stated on the manufacturers' websites. All homemade antibodies were validated on control and knockout mouse tissues in immunohistochemistry and on antigen-containing vs. non-containing extracts for all other applications.

Eukaryotic cell lines

Policy information about cell lines

Cell line source(s)	ATCC
Authentication	Neuro2a cells were authenticated by PCR.
Mycoplasma contamination	Neuro2a cells were not checked for mycoplasma contamination because they were only used for reporter assays after transient transfection.
Commonly misidentified lines (See ICLAC register)	N/A

Animals and other organisms

Policy information about studies involving animals; ARRIVE guidelines recommended for reporting animal research

Laboratory animals	Transgenic and control mice of both sexes were used in the study at embryonic ages and until the age of 5 months. All mice were on a mixed C3H x C57Bl/6J background. ARRIVE guidelines were observed.
Wild animals	N/A
Field-collected samples	N/A
Ethics oversight	Veterinäramt Stadt Erlangen, Regierung von Unterfranken

Note that full information on the approval of the study protocol must also be provided in the manuscript.

ChIP-seq

Data deposition

☒ Confirm that both raw and final processed data have been deposited in a public database such as GEO.

☒ Confirm that you have deposited or provided access to graph files (e.g. BED files) for the called peaks.

Data access links<br>*May remain private before publication.*	GSE119132 (https://www.ncbi.nlm.nih.gov/geo/query/acc.cgi?acc=GSE119132)<br>GSE119194 (https://www.ncbi.nlm.nih.gov/geo/query/acc.cgi?acc=GSE119194)
Files in database submission	For ChIP-Seq:<br>H2A.Z_SC_Ep400_ctrl_1.fastq<br>input_SC_Ep400_ctrl_1.fastq<br>H2A.Z_SC_Ep400_ctrl_2.fastq<br>input_SC_Ep400_ctrl_2.fastq<br>H2A.Z_SC_Ep400_ctrl_3.fastq<br>input_SC_Ep400_ctrl_3.fastq<br>H2A.Z_SC_Ep400_ctrl_4.fastq<br>input_SC_Ep400_ctrl_4.fastq<br>H2A.Z_SC_Ep400_cko_1.fastq<br>input_SC_Ep400_cko_1.fastq<br>H2A.Z_SC_Ep400_cko_2.fastq<br>input_SC_Ep400_cko_2.fastq<br>H2A.Z_SC_Ep400_cko_3.fastq<br>input_SC_Ep400_cko_3.fastq<br>H2A.Z_SC_Ep400_cko_4.fastq<br>input_SC_Ep400_cko_4.fastq<br>H2A.Z_SC_Ep400_ctrl.bigwig<br>input_SC_Ep400_ctrl.bigwig<br>H2A.Z_SC_Ep400_cko.bigwig<br>input_SC_Ep400_cko.bigwig

Genome browser session
(e.g. UCSC)

no longer applicable

Methodology

Replicates

For ChIP-Seq:
4 biological replicates for each genotype for H2A.Z-ChIP, sciatic nerve P9, mus musculus
2 biological replicates for each genotype for H3K27ac-ChIP, sciatic nerve P9, mus musculus

Sequencing depth

For ChIP-Seq:
- approximately 24 million reads per library
- 100bp length
- single-end

Antibodies

rabbit antiserum against H3K27ac (Diagenode, #C15410196, Lot#A1723-0041D, 1µg/IP)
rabbit antiserum against H2A.Z (Diagenode, #C15410201, Lot#A2039P, 1µg/IP)
control rabbit IgG (Sigma-Aldrich, #PP64, Lot#070M768V, 1µg/IP

Peak calling parameters

Reads were mapped to the mouse reference genome (GRCm38/mm10) using Bowtie (version 2.3.4.2, parameters: built-in-genome index, very sensitive end-to-end preset). For peak calling Model-based Analysis of ChIP-Seq (MACS2) (version 2.1.1.20160309.0) was used with input samples set as background and default parameters (q-value < 0.05).

Data quality

FastQC Read Quality reports (Galaxy Version 0.72) doing Basic Statistics regarding base sequence quality, sequence quality scores, base sequence content, base GC content, sequence GC content, base N content, Sequence Length Distribution, Sequence Duplication Levels, Overrepresented sequences and Kmer Content. Peaks after DiffBind analysis at 5% FDR: 2290, above 5-fold enrichment between ctrl and mutant: 1

Software

For ChIP-Seq:
- read mapping: Bowtie (version 2.3.4.2)
- removing PCR duplicates: RmDup (version 2.0.1)
- convertion to BAM format: SAMtools (version 1.1.2)
- BamCoverage tool within the deeptools software (version 3.0.2.0)
- peak calling Model-based Analysis of ChIP-Seq (MACS2) (version 2.1.1.20160309.0)
- visualization: Integrated Genome Viewer (IGV; version 2.3.98)
- heatmaps and plotprofiles: deeptools software computeMatrix scale region mode (version 2.5.0.0)
- differential binding analysis: DiffBind (version 2.6.6)
- intersection with genes: intersect (version 1.0.0)
- connection of peak lists: join (version 1.0.0)
